# Supplementary figures and images for: Transcriptomic and coexpression network analyses revealed the regulatory mechanism of Cydia pomonella infestation on the synthesis of phytohormones in walnut husks
Source: PeerJ. 2024 Sep 23;12:e18130. doi: 10.7717/peerj.18130 (PMC11426320; doi:10.7717/peerj.18130)

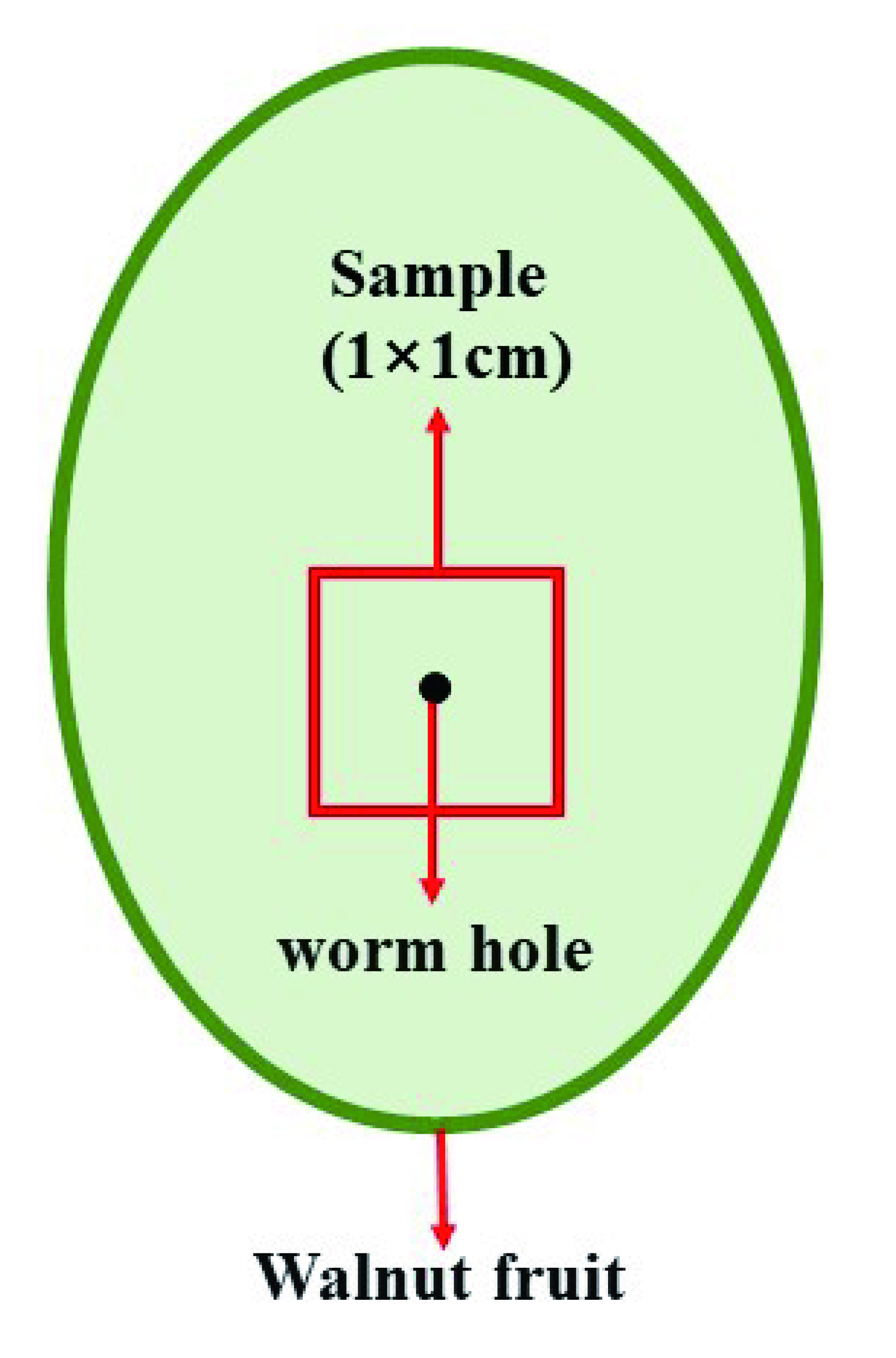

Supplement: Supplemental Information 1 [file peerj-12-18130-s001.tif]

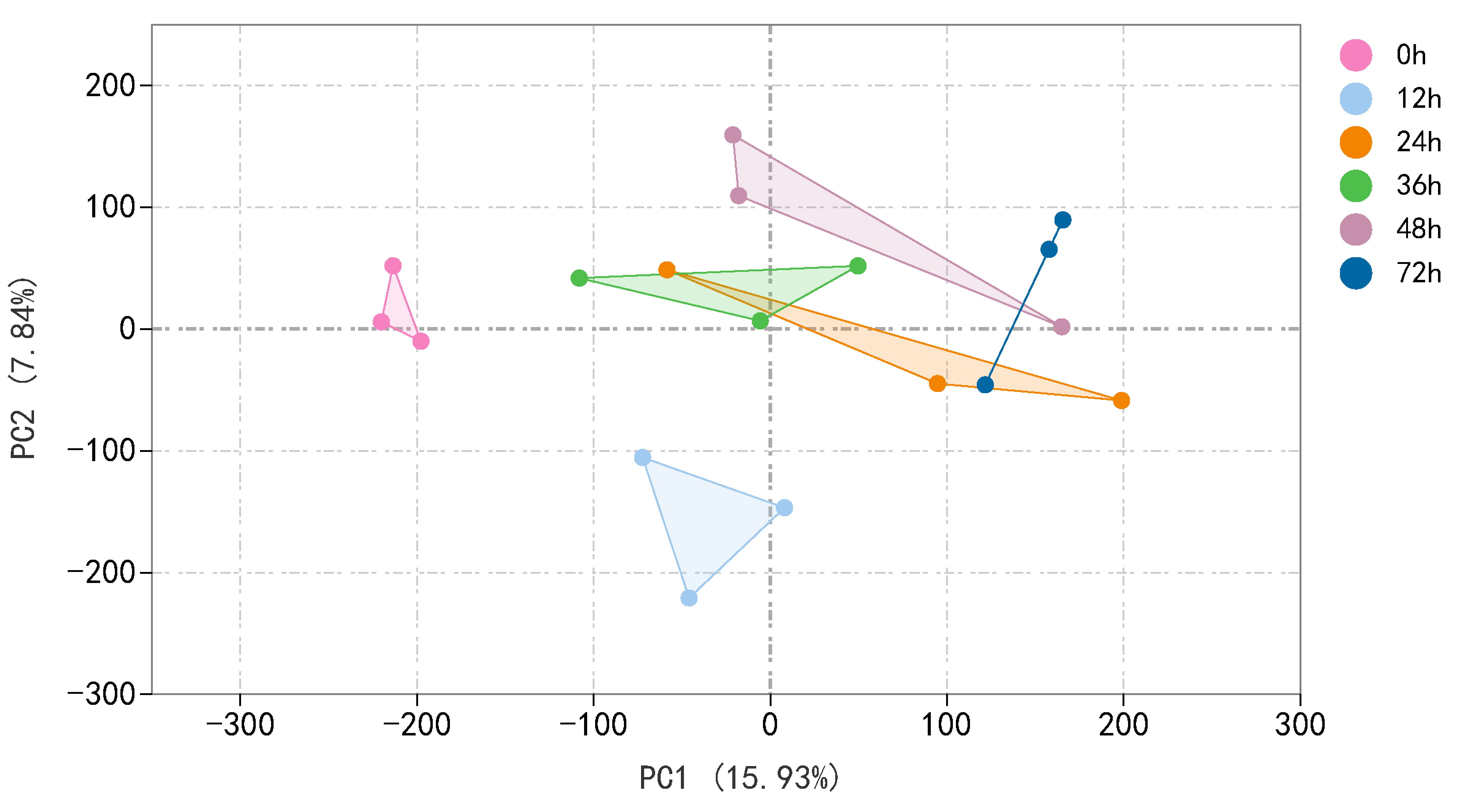

Supplement: Supplemental Information 2 [file peerj-12-18130-s002.jpg]
